# Considerations for Meeting Students' Mental Health Needs at a U.S. University During the COVID-19 Pandemic: A Qualitative Study

**DOI:** 10.3389/fpubh.2022.815031

**Published:** 2022-02-02

**Authors:** Jessalyn Kaur, Eva Chow, Johanna Ravenhurst, Teah Snyder, Sheila Pennell, Andrew A. Lover, Sarah L. Goff

**Affiliations:** ^1^Department of Health Promotion and Policy, School of Public Health and Health Sciences, University of Massachusetts Amherst, Amherst, MA, United States; ^2^Department of Epidemiology, School of Public Health and Health Sciences, University of Massachusetts Amherst, Amherst, MA, United States; ^3^Elaine Marieb College of Nursing, University of Massachusetts Amherst, Amherst, MA, United States

**Keywords:** COVID-19, university students, qualitative, mental health, recommendations

## Abstract

**Objective:**

To better understand the potential ongoing effects of the COVID-19 pandemic on U.S. university students' mental health and to generate hypotheses as to how universities may best meet students' mental health needs.

**Participants:**

Students at a large public university in the United States.

**Methods:**

Students were invited *via* email to participate in either interviews or focus groups regarding their COVID-19 pandemic experiences, including mental health impacts. Recruitment took place in two waves. Sessions were led by student members of the research team and took place *via* video conference between December 2020 and June 2021. Interviews were audio-recorded and transcribed *via* Zoom and manually edited and de-identified by the research team. Interview data were analyzed deductively and inductively using a modified grounded theory approach.

**Results:**

A total of 40 undergraduate and graduate students took part in the study. Major themes included: (1) Overall impact of the pandemic on mental health; (2) Sources of pandemic stress/mental health impacts; (3) Subsequent coping strategies; and (4) Suggestions for improving university support for student mental health. Subthemes were identified within each major theme. Students reported substantial anxiety and other mental health impacts and felt improvements in communication and access to mental health resources could better support students.

**Conclusions:**

This study provides context for the ways in which the COVID-19 pandemic may be continuing to impact mental health in a north-American university setting and identifies suggestions for potential interventions that future studies may test for effectiveness.

## Introduction

Since the SARS-CoV-2 virus was first detected in Wuhan China in late 2019, countries across the globe have episodically imposed mitigation measures such as restrictions on public gatherings, lockdowns, mask mandates, social distance mandates and travel bans, in an effort to stem the rapid spread of the virus [(1–3); CDCMMWR]. These mitigation measures started in full force in mid-March of 2020 in Massachusetts (MA), a state in the northeastern U.S. The measures included sending all college and university students home, where many would remain until the Fall semester of 2021. Ultimately over 850 million students worldwide transitioned to alternative teaching methods (4). The abrupt closure of campuses, followed by substantial societal changes, presented and continue to present challenges for university students who not only must navigate a global pandemic but must also adjust to studying independently (5).

Isolation and other pandemic stressors have increased students' risk for anxiety, depression, and other mental health disorders. Studies conducted in the earliest days of the pandemic identified increased levels of anxiety, depression and stress (6–9). More recent studies have identified ongoing stress, anxiety and depression, including negative impacts of online learning on mental health and differences in mental health impacts based on sex and race/ethnicity (10–12). The majority of studies describing the mental health effect of the pandemic on university students' have used quantitative methods such as surveys. Gubrium and Gubrium described the importance of narrative methods to understand human impacts of an unprecedented global health event (13) yet there have been few narrative studies seeking to better understand university students' experiences with mental health during the pandemic or perceptions of university response to the pandemic and support for mental health.

This study used narrative methods to begin to address these gaps in knowledge. The study's aims were to gain a deeper understanding of students' lived experiences at one U.S. university in relation to mental health during the pandemic and to explore their experiences with perceived university support for student mental health. The study results could inform future studies that aim to develop and test the effectiveness of interventions to support university students' mental health. The COVID-19 literature continues to evolve rapidly and this manuscript represents the information available at the time it was written; new data may emerge in the ensuing review period.

## Methods

### Study Population and Recruitment

Two waves of qualitative data collection were undertaken at a large public university in the northeastern US. In Wave 1, focus groups and interviews were conducted with eligible students to explore their experiences with the pandemic and with the university's response to the pandemic. Data were collected between December 2020 and January 2021. For the focus groups, a random sample of 1,130 undergraduate students and 296 faculty, staff, and graduate students were invited *via* e-mail to participate. Students were eligible if they were 18 years of age or older and lived in the university area during the fall 2020 semester or planned to live in the local area during the spring 2021 semester. Anyone in the random sample who had been contacted by the university for contact tracing purposes was excluded from the focus group recruitment emails. A total of 400 members of the university community who had been directed by the university's COVID-19 contact tracing program to either quarantine or isolate were invited *via* email to participate in individual interviews rather than focus groups to maintain confidentiality about COVID exposures or infections. All participants received a unique link to a secure RedCap project that included an eligibility survey, an electronic informed consent document explaining the study, and a brief demographic survey (14, 15).

In the second wave of data collection, undergraduate and graduate students affiliated with the university's school of public health were invited *via* e-mail to participate in interviews regarding their COVID-19 pandemic experiences, including mental health impacts. Participants were eligible to participate if they were 18 years of age or older and currently enrolled at the university. Interview sessions were led by student members of the team and took place *via* video conference between April 2021 and June 2021 using pre-tested interview guides. Between 20 to 40 interviews were estimated to be needed to achieve data saturation based on the homogeneity of age and life stage among participants and the narrow focus of the study. This estimate was based on recommendations in methodological texts and papers as well as publications of similar studies.in journals with high impact factors (16–20).

Participants in both waves of the study provided verbal consent to participate in the study and to have the session audio-recorded. If a student did not want to be recorded, detailed field notes would be taken. At the beginning of each session for both waves, consent documents were explained and questions were answered. Protocols for both waves of the study were reviewed by the university's Institutional Review Board (IRB#1873 and IRB #2003) and determined to be exempt from the federal regulations that govern human subjects research.

### Wave 1 Interviews and Focus Groups

Interview guides for both the focus groups and the individual interviews were developed and pre-tested by the research team. The guides consisted of open-ended questions followed by probes that targeted areas of interest, such as experience in isolation or quarantine, the university response to the pandemic, and the impact of the pandemic on students' lives (Supplementary Appendix A). One of the study's primary aims was to explore health behaviors and decision making-processes related to COVID-19 for students who had been exposed to or contracted COVID-19 and those who had not. Mental health was an important element of this exploration.

Focus groups were facilitated by two graduate students and one undergraduate student trained in qualitative research methods (TS and JR). Students led the focus groups to increase the likelihood of discussion of behaviors that may not have been consistent with university guidelines. SG and AL provided guidance on facilitation methods and the research team met regularly to discuss study progress. A maximum of nine participants were invited to each student focus group. Focus group sessions lasted ~90 mins. Participants received a $10 Amazon e-gift card.

Individual interviews were conducted by a clinically trained faculty member (SP) affiliated with the university's Public Health Promotion Center to maintain student confidentiality. The consent form was reviewed with an opportunity for questions and verbal consent confirmed at the beginning of each interview. The average time for each interview was 45–60 mins. Participants received a $10 Amazon e-gift card.

The interviews and focus groups were conducted over Zoom and recorded with participant permission. Zoom-generated transcripts were reviewed by research assistants to correct transcription errors and to remove identifiers.

### Wave 2 Interviews

For Wave 2, the focus group interview guide was adapted to increase focus on mental health and university communications, drawing on themes that emerged in Wave 1, Existing literature on student mental health, student investigator experiences, and study aims informed interview guide development. The guide was tested for clarity and completeness with a sample of students serving on a COVID communications committee (Supplementary Appendix B). The guide consisted of open-ended questions followed by probes that targeted areas of interest such as, the aspects of the pandemic participants found most challenging, silver linings, loneliness, sadness/depression, family members at risk, and thoughts and opinions on the university's response. The students who conducted the interviews were trained in qualitative interview skills by an experienced qualitative researcher (21–23). As the interviews were conducted, the interview guide's probes were amended in an iterative process as new concepts emerged. Interviews took place *via* Zoom video conference between April 2021 and June 2021 using pre-tested interview guides. Interviews were audio-recorded and transcribed *via* Zoom transcribing service. A consent form containing detailed information regarding the study was given to all participants and reviewed with participants prior to their interview. Interviews were conducted until data saturation was achieved (24, 25), defined as no new concepts emerging over at least three consecutive interviews. Participants received a $5 gift card to a coffee shop as compensation.

## Analysis

Data were analyzed both deductively and inductively using qualitative content analysis (26), a subset of the broader umbrella of thematic analysis (27). The following overlapping analytic steps (spiral analysis) were taken: (1) Preparing and organizing the data for analysis, including transcription of audio recorded data; (2) Becoming familiar with the data; (3) Memoing the data; (4) Coding the data; (5) Moving from codes to categories and categories to themes. JR and TS led the analysis of Wave 1 data. They first reviewed field notes and transcripts of focus groups to fully familiarize themselves with the data, creating memos to capture emerging thoughts about patterns in the data. A preliminary code book was created during this process based on interview questions and memos. Transcripts were uploaded to Dedoose, a qualitative software platform (Version 9.0.17, 2021, Los Angeles, CA). JR and TS then independently coded two focus group transcripts and met to compare codes and resolve discrepancies through consensus. The code book was refined in an iterative process during this phase of analysis. AL, SG, SP participated in a subset of analytic meetings to aid in the resolution of coding discrepancies, refinements of the code book, and grouping of codes into major themes and sub-themes, connecting them back to the data and identifying illustrative quotes. Sorting of codes into categories then to themes took place in an iterative process throughout the analysis. Reflexivity considerations were discussed during and across the analysis spiral. This analytic process was repeated for Wave 1 interview data, with minor revisions to the code book, themes, and sub-themes emerging.

Analysis of Wave 2 interview data proceeded similarly to the approach used for Wave 1. All of the students participating in the analysis had been previously trained by SG in qualitative analytic methods. The four students (JK, SC, EC, MF), who led the interviews reviewed the transcripts to familiarize themselves with the data, to correct transcription errors, and to generate memos and develop a preliminary code book. The Wave 1 code book and interview questions were also used in the development of the preliminary code book. Using the provisional code book, two teams of two coders independently coded the first two transcripts using Dedoose software. Teams then met to compare and adjudicate their code selection, followed by full team meetings to resolve any differences in opinion regarding code application and to identify and discuss categories and themes emerging from the data. This process continued with ongoing memoing and the code book being amended as new categories and themes arose during the coding process. As the code book was refined students went back to previous transcripts in an iterative process to revise the coding of transcripts using the most up-to-date code book. Reflexive considerations included close interactions between the student researchers and direct subject matter expertise and SG's secondary role as a primary care clinician who cares for college-aged patients.

## Results

A total of 40 students took part in the study, including 13 undergraduate students who participated in one of four focus groups, 11 undergraduate and graduate students who participated in Wave 1 interviews and 16 undergraduate and graduate students who participated in Wave 2 interviews. Eight participants self- identified as male; 25 as female; one as gender diverse, and one as non-binary (2.5%); five participants did not provide information regarding their gender (12.5%). All participants were at least 18 years of age. In regard to race, 21 people identified as non-Hispanic white; three as Hispanic/Latinx; seven as Asian, two as Asian and White; one as Black and one as Asian and Hispanic. Five people did not provide information regarding their race/ethnicity. Six of the participants were graduate students and the other 34 participants were undergraduate students. Participant demographic characteristics can be found in Table 1.

**Table 1:** Participant demographic characteristics.

**Participant characteristics (*n* = 40)**	***n* (%)**
**Self-identified gender**	
Female	62.5 (25)
Male	20 (8)
Non-Binary	2.5 (1)
Gender Diverse	2.5 (1)
Not disclosed	12.5 (5)
**Self-identified race/ethnicity**	
Caucasian/White	52.5 (21)
Hispanic/Latinx	12 (3)
Asian/Asian-America	17.5 (7)
Black/African American	2.5 (1)
Mixed race Asian and Hispanic	2.5 (1)
Mixed race Asian and Caucasian/White	5 (1)
Not disclosed	12.5 (5)
**Education level**	
Graduate student	15 (6)
Undergraduate student	85 (34)

Four major themes were identified, including: (1) overall impact of the pandemic on mental health; (2) coping strategies; (3) perceptions of university support for mental health; and (4) stress. Each theme had associated sub-themes which are described in detail below with supporting quotations. Participant gender, race, whether they were a graduate or undergraduate student, focus group or interview participant, and interview identifier are provided with each quote. Table 2 provides additional supporting quotations for each theme.

**Table 2:** Themes and subthemes with supportive quotations.

**Themes and subthemes**	**Quotations**
**1. Direct mental health impacts due to the pandemic**	
Anxiety/Worry	“Anxiety, constant, about anything. I guess, even seeing one person, socially distant, masks on, outside, was terrifying to me for a long time, up until I got vaccinated and we started figuring more out about the virus…” (Female, White, Graduate,Wave 2 Interview #8)
Loneliness/isolation	“Definitely loneliness because like you're not with your friends as much as you used to be or, you know, like a lot of people, like a lot of my friends. When I moved back home, a lot of them wanted to stay socially distant and stuff, and like so did I for a while but then like, which I totally understand but it's just like, it's so hard to be alone for so long, like I had my family obviously but you don't always want to be with your family 24/7 so it did get kind of lonely at times” (Female, White, Undergraduate, Wave 2 Interview #16)
Precarity	“The pandemic itself kind of brings its own kind of change in mindset, I believe, especially for being in college, like, I went from being like a sophomore and still having 2 years of college, and then the pandemic hit and made me think of like what am I going to do when I graduate because like the, because like internships are harder to find and like jobs are kind of harder to find in general so it kind of like pushed me forward a bit like, okay, like, what's the future looking like? (Non-binary, White, Undergraduate, Wave 2 Interview #11)
Frustration	“But at the point we're speaking to some people it's been 2 weeks, since they got that test, and for them to have to think about who they've been in contact with 2 weeks ago, those people are already testing positive and already spreading it. And so, it felt very futile, it was unbelievably frustrating, because the system was just so slow” (Female, Asian, Undergraduate, Wave 2 Interview #9)
Mental Health challenges prior to the pandemic	“Very anxious person, and I feel horribly with like last minute changes. So, to have last minute changes thrown at me so many times throughout the year was a growing experience, but it sucked. It was hard” (Female, White, Graduate student, Wave 2 Interview #5). “I definitely consider myself someone who has levels of social anxiety. So coming into this like I've had familiar feelings with like being really nervous about things that kind of dwelling on things like ruminating thoughts” (Female, Hispanic/Latinx, Undergraduate, Wave 2 Interview #8)
**2. Sources of pandemic stress/Mental Health impacts**	
Academic sources of stress	“So many students, especially STEM students feel very behind because you're supposed to be getting into these labs, you're supposed to be doing all this experience and it's just not the same, and there's just no forgiveness.” (Female, Asian, Undergraduate, Wave 2 Interview #9)
“Big picture” sources of stress	“I guess mental health wise that's a totally other aspect with all of the panic and paranoia and everything with the news and media and the election which was such a dark time for everyone, but it was incredibly hard to face everything in my bedroom, which is where I was facing everything at the exact same” (Female, White, Graduate Student, Wave 2 Interview #10)
At-risk Family member	“I think I'm going off of what I just said to look at Asian hate crimes. I was really stressed about my family members, getting like hurt, or getting COVID, because I do have some older family members I'm really close to as well as like my little sister has. I think it's called neutrophillia so she doesn't have as many white blood cells so she's pretty immune to getting like a lot of like getting really sick. So, I was just really stressed out, especially since this spring. I was on campus, so I wasn't home” (Female, Asian, Undergraduate, Wave 2 Interview #6)
Stability of relationships	“Yeah definitely on my mind all the time I know that it was definitely, like I didn't see friends and I had, I saw my significant other, primarily it's just like him and then the person I lived with my mother. So it was like kind of balancing that like I will still see him, because that's kind of like, it was like the social pod I created” (Female, Hispanic/Latinx, Undergraduate, Wave 2 Interview #8)
Direct stress due to COVID	“I'm relatively young and I'm generally healthy. On the other hand, I'm autistic which significantly increases my chance of severe COVID-19. Um, I- given the statistics, I would estimate that I have a pretty high chance of never having symptoms or having relatively mild symptoms, but a significant chance of having lasting or lasting symptoms that could be a major problem for me. A close friend of mine, for example, has pretty much had COVID symptoms since summer and is now having to be tested for fibromyalgia” (Male, White, Undergraduate, Focus Group #2)
	“I didn't really know how to control, like you know the anxiety and the stress of not only my academics, but mixed with like potentially having COVID, because even like when I left isolation, they said like. Like yeah, you're not contagious. But you might like, in the next 90 days like you'll be positive, and like you might develop symptoms any time. Like, because we don't have enough data, like you might develop symptoms in the next 90 days, which is scary because I'm just like, Okay, well now I have to, like, you know, like think about this for the next 90 days” (Undergraduate student, Wave 1 Interview #5)
Financial stressors	“I also feel like it's really hard right now for people to do anything without prioritizing financial compensation just because it is a financially difficult time” (Undergraduate, Focus group #4)
Compounding stressors	“… your future is riding on how well you do in these classes and thinking about that, you're also worrying about your family at home and you're worrying about your safety and I'm also working a job, so I'm worrying about that. And am I getting enough money because I need to pay for my apartment so that I can live in Amherst because I got fired as an RA and so now, I need to work this jobs that I can pay for this apartment. And are my friends safe” (Female, White, Undergraduate, Focus Group #4)
**3. Coping strategies**	
Self-care	“I think the hardest part about all this is having patience for myself because there's so there's such a lack of empathy, compassion and patience in every aspect of our society at this current moment. So at least I can offer that patience for myself” (Female, Asian, Undergraduate, Wave 2 Interview #9)
Professional mental health services	“Yeah, but I don't know, I don't really I have never tried going to therapy. I don't think it's a bad idea I just don't really know where to start with it because before I was I never had any mental health issues” (Female, Asian, Undergraduate, Wave 2 Interview #6)
Setting Boundaries	“So actually my second semester I decided to do a part like part time so I only did two classes, because that worked out with my schedule so I could still graduate on time if I did that. Although my senior year probably will be harder. And I decided to work part time at a preschool I worked in the summer, which I wouldn't have done without COVID. So, yeah, I could like, it completely changed I guess my academic… because I never would have gone part time if it wasn't for COVID (Non-binary, White, Undergraduate, Wave 2 Interview #11).
Social Support	“… yeah so that definitely helps, staying connected with friends and family *via* zoom or FaceTime or WhatsApp. And that's actually another thing that we learned in the course, the importance of socializing with people and how that also helps us destress” (Female, White, Undergraduate, Wave 2 Interview #12)
	“It was nice. I think the only thing that really saved me and kept me grounded during that time was. I was living with my boyfriend and my family and Tennessee. But if I was alone, I don't know, that would have been a very dark time, perhaps I would have dropped out or something. (Female, Asian, Graduate Student, Wave 2 Interview #4)
**4. Perception of university support for mental health and recommendations**	
University communications	“But, yeah, just like reiterating what I all said, I think that *** could definitely do a better job at showing students that they care and that mental health matters.” (Female, Asian, Undergraduate, Wave 2 Interview #6)
	“They also would send out emails to us every once in a while with informing us about counseling services that the university offers, if you're feeling stressed” (Female, White, Undergraduate, Wave 2 Interview #10).
University resources	I know that ^***^ had like team positive presence and stuff like giving free things around, and they were like little activities outdoors and stuff toward the end of the semester, which I think is nice.
	I think it could have happened sooner, to, yeah, just like, definitely, including those resources like at the bottom of every email like they don't even need to acknowledge it, just like put at the bottom that email and say like the name and stuff would be nice to like so you can't really miss it. (Female, Asian, Undergraduate, Wave 2 Interview #6).
Support from professors	“And like when I got home. I liked email my professors and they were also so great. I was also nervous. Like, I don't know if they were going to be like oh so clearly, you're seeing people.” (Female, White, Undergraduate, Wave 1 Interview #9)
Suggestions for improving mental health services within the university community	“Yeah, again, like, um, the school like they should notice that like, students really receive many emails from like different departments. So really try to like make the emails, if they want people to like actually read them like for effective. Like, definitely 1 can consolidate like make, condense the message, make it like easier to read. And like, if it's any like, important, important message that they do, make it look like, like uh, make it yeah pop out. (Female, Asian, Undergraduate, Wave 2 Interview #14)

### Theme 1: Overall Impact of the Pandemic on Mental Health

The pandemic led to many sudden changes to university students' lives. Not only were students tasked with completing classes and schoolwork at home, but they also had to process the changes brought on by the pandemic such as mask and social distancing mandates, potentially impacting mental health. The major theme “overall impact” included subthemes related to specific symptoms, feelings, and conditions that participants linked to the pandemic. An additional subtheme describes the effect of the pandemic on participants who had mental health conditions prior to the pandemic.

#### Anxiety/Worry

When asked about anxiety/worry during the pandemic many students commented on situations that made them more anxious or worried. Several students discussed long-term anxiety throughout the duration of the pandemic. For example, one student found that the anxiety they experienced was “constant” and that many aspects of the early pandemic such as going “outside” was “terrifying”. (Female, White, Graduate, Wave 2 Interview #10).

Another student commented on one of their first experiences going to a grocery store once the pandemic started:

“I had so much anxiety around it like going to the grocery store I remember one of my first experiences I had like after it was established that it was in the US and it was very serious, I went to the grocery store and I wore gloves because no one's wearing masks yet but I was like okay I don't want to touch anything and like transmit anything so I wore plastic gloves.” (Female, Hispanic/Latinx, Undergraduate, Wave 2 Interview #8).

#### Loneliness/Isolation

Many students realized just how much a loss of social interaction was impacting their mental health. Due to various stay-at-home orders and online schooling, many students found themselves feeling lonely or isolated. Some international students had to cope with flying to their home country and having to enter a prolonged isolation period:

“I feel like the most difficult is that when I flew back to Tokyo I have to like, stay in my own place for an entire 2 weeks, I know like, the United States don't really require people to like mandatory, mandatory stay on their own place to quarantine when that flew back from another country. It is how it is in Japan, so it gets really lonely for the entire 2 weeks, or not be able to contact with an actual human, including my family” (Male, Asian, Undergraduate, Wave 2 Interview #15).

#### Precarity

When the pandemic first reached the U.S many things were unknown. As time goes on we are still learning about the virus. The lack of knowledge about COVID has left many people to question what the future will look like in the coming months. Several students spoke about their uncertainty regarding the future after COVID. One student spoke about the loneliness they felt when the pandemic first hit, describing it as a sudden “big change.” They continued to describe the uncertainty they felt regarding the future:

“And then suddenly having the pandemic hit and then suddenly I'm like, alone, basically, I mean with my family but like, no friend interaction, like so suddenly is definitely a big change. And I think it was kind of the change, not even that like, it was suddenly a big change, but it was a big change and then I didn't know what was going to happen next because like, when we left we were, I think when the pandemic hit we're leaving for spring break, and then they're like, oh, you'll only be out for the rest of the semester.” (Non-binary, White, Undergraduate, Wave 2 Interview #11).

Another student discussed the sense of fear they had about the future after the pandemic:

“But just kind of like existential dread, thinking about what life could possibly be like after this and how a pandemic is only once in a generation type of thing, like we go on the we go on the subways and public places with a crowd of people all the time I just, it makes me so nervous to try and start going back to normal, with things lifting up soon. Even though I'm fully vaccinated I, I almost don't feel safe taking off my mask.” (Female, White, Graduate student, Wave 2 Interview #10).

#### Frustration

COVID-19 was not only new and unknown but it led to changes in the way that we interact with each other and the world. With the shift to online schooling as well as policies put in place by the university and the government, many students became frustrated with the situation that was forced upon them. One student discussed their frustrations regarding the way online learning has impacted their academics:

“Because I'll be honest like freshman year before COVID hit. I would, I was doing the work you know I was actively trying to make sure I was learning, but now it's just like I look at the book, I spit out the information I get the grade rinse and repeat over and over again, and I don't want to keep doing this” (Female, Hispanic/ Latina, Undergraduate, Wave 2 Interview #7).

Students also discussed frustrations they felt by actions taken by the university to address the pandemic:

“And then eventually being told like in the fall semester that also we as Contact Tracers cannot talk about not just like specifics because obviously HIPAA, but what groups on campus [had high rates] with the university is saying, the average amount of cases we weren't allowed to say any of that until the university said anything, and that was unbelievably frustrating because not only am I having to deal with these people, I can't even voice, what is happening. And that felt really disrespectful” (Female, Asian, Undergraduate, Wave 2 Interview #9).

Participants also discussed their frustrations with the U.S government's approaches to the pandemic:

“I think the most important thing is none of this was necessary And that's what I think people forget about all the time. Is this the result of incompetence and showboating, and the prioritization of money and profits. No one had to die like this, and no I should not have had, like I'm probably gonna have to go to therapy for years because of this. There's real costs to these decisions, and I hope as a country, and as a global community, that will never be forgotten. And I hope 1 day accountability will take place, but I hold out hope” (Female, Asian, Undergraduate student, Wave 2 Interview #9).

#### Mental Health Challenges Prior to the Pandemic

Some students discussed how the pandemic has impacted them and their prior mental health conditions before the pandemic. One student found that the pandemic worsened all of their current mental health conditions.

“And I currently have anxiety I currently have depression. I currently have OCD as well and all of those have been worsened and by the pandemic” (Female, Hispanic/ Latina, Undergraduate, Wave 2 Interview #7).

### Theme 2: Sources of Pandemic Stress/Mental Health Impacts

With the changes that came with the pandemic, many students expressed feeling stressed. This theme explored the various sources of stress students reported which are identified as subthemes.

#### Academic Sources of Stress

As the university switched to online learning many students felt increased stress juggling the rigor of classes as well as the changes in material and exam delivery. As one student said:

“I'm trying to track what assignment to do across like five different services. It's hard enough across one service, I've forgotten several entire tests. It's just not workable, it's hard enough to do that in person and it's much harder when I have to remember to check all these different things when I might not have been in class, it might not have even been mentioned, even if I was in class, etc. (Male, White, Undergraduate, Focus Group #4).

#### “Big Picture” Sources of Stress

COVID-19 brought about many changes not only to academic life but to general society. There were various “big picture” sources of stress students discussed such as the politicization of COVID, general mistrust and impacts on vulnerable populations. One student commented on the politicization and mistrust surrounding COVID-19:

“And so I think it's just kind of made me like more stressed than normal because there are so many people who have come up with like conspiracy theories about the pandemic and there's so much like mistrust in people and the vaccine things like that so it's been really kind of stressful to see where certain people stand on it and seeing like just the amount of hate that's kind of come out of it and things like that. And so it's caused a lot of stress because it's become so political and it's not just about public health anymore.” (Female, White, Undergraduate, Wave 2 Interview #16).

Another student discussed how the recent U.S presidential election added to the stress revolving the pandemic:

“I guess, all of the politics, graduating, going to a new space, having to grow up a lot faster than I wanted in that last year was incredibly difficult because I had to kind of think about what the election meant and stuff to me, talk to my friends over text rather than seeing them in person.

So it was pretty hard to, I guess, find a place in the world, after graduating and being a freshman at a new college that has like 30,000 kids that I haven't met yet, was really difficult.” (Female, White, Graduate, Wave 2 Interview #10).

#### At-Risk Family Member

Another major cause of stress for students came from fear that their family members who were more at risk for severe infection from COVID-19 would get the virus.

“And I guess it was just the dread of my parents ever getting COVID because they're not they're not young but they're not old and I wouldn't, I wouldn't be able to fathom them getting it.

Even if I did, which I really didn't care about. So, yeah, just dread and the fear and the anxiety of them.” (Female, White, Graduate, Wave 2 Interview #10).

#### Stability of Relationships

With stay at home orders in place and the university no longer holding in person lectures during the Spring following the arrival of the pandemic, some students worried about the impacts that not seeing their friends would have on their relationships. One student who lived near her friends elaborated on what they feared would happen if they weren't able to physically see their friends:

“If I was at home when I didn't get the opportunity to live near my friends, I probably would have lost communication, not entirely but for the most part of it. And that would have been a big stressor because I felt like, I would feel like a burden on them if I wanted to, like, you know, like talk to someone, because we wouldn't be as close.” (Female, White, Undergraduate, Wave 2 Interview #13).

#### Direct Stress Due to COVID

As the number of cases and hospitalizations due to COVID grew so did the amount of fear and stress people experienced due to COVID-19. Many students discussed the fear they had of receiving the virus due to pre-existing conditions or from personal experiences with a friend or loved ones who were negatively impacted due to the virus. The two quotes below showcase the various reasons why the fear of getting COVID-19 induced a stress response:

“I would definitely be pretty stressed out if I tested positive because I know somebody who is similar in age and he was hospitalized and has like muscle wasting, severe muscle wasting. He was extremely fit prior. So, I don't think it's something that doesn't affect our age group” (Female, Hispanic White, Undergraduate, Focus Group #2).

#### Financial Stressors

Students discussed the financial impacts they experienced due to the pandemic and how that impacted their mental health:

“Yeah, and I guess there was also a little bit of like a financial stress because, it made my father's company do worse, because of like the whole COVID … now it's fine but like back in like the fall semester, I think that was like a real stress.” (Non-binary, White, Undergraduate, Wave 2 Interview #11).

#### Compounding Stressors

Often times the students did not just have one source of stress but had many different factors that piled onto each other creating compounding stressors. For example, one student said:

“In general money and like paying for rent, I was obviously off-campus, so making sure I wasn't spending a lot of money, and school in general because we didn't get any breaks, like we never got a break. It was just like piled on and on.” (Female, White, Undergraduate, Wave 2 Interview #13).

Another student described the Fall semester during COVID as the “hardest semester” of their life:

“It's definitely this past semester has been the hardest semester of my life, for a lot of reasons, both like personal ones and academic wise and at the start semester I was working 20 h a week, I was trying to…. I work for a student business, my co-workers basically abandoned ship and just left it to me. And one other co-manager who was going to be graduating, and so that pressure was unbelievable. And then, you know, I'm trying to do my school work. I'm trying to get all this other stuff in order, run a club…I'm a president of a club and trying to do that… and then also just be a person, you know I like cooking, I like hanging out with my roommates and I, at first didn't have time for that and so I had to realize that I needed to cut back” (Female, Asian, Undergraduate, Wave 2 Interview #9).

### Theme 3: Coping Strategies

The sudden changes brought on by the pandemic led some people to find ways to take care of their mental wellbeing. Students reported coping strategies largely related to self care, social connection and professional mental health services.

#### Self-Care

Many students engaged in various types of self-care as a way to take care of themselves and their mental health:

“I've gotten really into like routine of exercising outside, which I think has been very helpful because if not, I would have just sat my room all day, doing school stuff and then other projects, etc online. So I think definitely seeking out things like that outside is really important and then maybe if you can't see friends or family or loved ones kind of just picking up like a hobby or something that kind of is fulfilling and might like even be like, like reward based like I'm thinking about growing a small garden this summer. So playing with that idea or even like reading a book that's like completely for free reading has been very helpful like just totally kind of wipes your mind a little bit.” (Female, Hispanic/Latinx, Undergraduate, Wave 2 Interview #8).

#### Professional Mental Health Services

A few students discussed reaching out to professional mental health services like a therapist in order to take care of their mental health during the pandemic:

“I guess I've been forced to kind of look at my mental health, because I've been, spending a lot of time in my room alone for the entire year, and I was able to get more appointments with a therapist to talk about anxiety and paranoia and stuff like that. Yeah, and I think I'm going to continue to do that, even afterwards and just, there's a there's a stark before and after for my anxiety and mental health, and I think even though it did get worse and it's pretty bad, I'd see that it's going to get better as soon as things start opening up because I know how to, how to deal with the bad.” (Female, White, Graduate Student, Wave 2 Interview #10).

#### Setting Boundaries

One way students were able to cope with the pandemic was by creating boundaries for themselves in order to prioritize their mental health.

“I instead was like okay I'm just gonna work to make my life easier. So, I have time to work on my mental health with the skills I've learned from being in therapy before because luckily I have like, saw a counselor before and, and it's gone well. And so that's why I lowered the numbers of hours. I reached out to the woman in charge of the Center for Student business and was like, I can't do this, this is how my co-managers are treating me.” (Female, Asian, Undergraduate, Wave 2 Interview #9).

#### Social Support

Many students looked to their friends and family as a source of support to relieve stress.

“So it was nice for me to like leave the desk, and like go see other people, like if I didn't do that like, if I didn't really like engage in like an outside activity and put myself out there probably would have been in a dark hole.” (Female, White, Undergraduate, Wave 2 Interview #13).

### Theme 4: Perception of University Support for Mental Health and Recommendations

When asked about the university's response to COVID in the context of student mental health, some students discussed the ways in which the university communicated mental health resources and the utility of those resources. Students discussed their views on how they felt the university supported them and their mental health during the pandemic. Participants discussed the various ways in which the university provided support for students and the ways in which the school could improve on supporting students.

#### University Communications

The views on the university's communication to students regarding mental health were mixed. Many students felt that the university did not reach out to students effectively regarding mental health support:

“But in terms of mental health outreach its was lacking. Like if I'm being honest I rarely saw any emails in my inbox or any posts on the Instagram account where is was like ‘hey these are the resources we have, these are some tips to help you out a little bit. All we really got were two Wellness Wednesday's that weren't really Wellness Wednesday's and that's about it.” (Female, Hispanic/Latinx, Undergraduate, Wave 2 Interview #7).

While many students found the universities communication could be improved others thought that the university did an efficient job in communicating the importance of mental health:

“And even difficulties with mental health during the pandemic like they've been really on top of that so I appreciate it.” (Female, Asian, Graduate, Wave 2 Interview #4).

#### University Resources

During the interviews students commented on the accessibility, quantity and quality of mental health resources provided by the university. Some students found the school provided a “variety of resources” but they may not be used by all students:

“But I guess it's not anyone's fault that it's hard to like, make the school, like reach out for those in need, who don't look for resources, because you never know. Okay, like that's always a challenge. And not everyone is brave enough to like, look for these resources, or maybe some people might not even realize they need these resources. But I think in general the school really provides a lot of variety of resources definitely.” (Female, Asian, Undergraduate, Wave 2 Interview #14).

Other students found that the mental health resources provided by the university were not adequate:

“I eventually reached out to the ^***^ at the Center for Counseling and Psychological Health or something, I don't know. Yeah, but I reached out to them. I was very unsatisfied with the care they gave me…She gave me two therapists, I made it very clear I did not have transport, I just take the bus everywhere I walk. And she gave me two therapists which were not in the local area. They didn't have any information online, and I was just really frustrated by that. And it felt very much like… because they knew there was a therapist shortage which also sucks for everybody.” (Female, Asian, Undergraduate, Wave 2 Interview #9).

#### Support From Professors

Respondents reported a very wide range of experiences in their interactions with faculty. Some students found that their professors were understanding regarding their situation:

“It can feel really hard like you can feel like you're on your own, when you're doing online classes and like some professors are really good at like making an online presence and giving a lot of students like opportunities.” (Female, White, Undergraduate, Wave 2 Interview #16).

However, students felt unsupported by professors due to a lack understanding or perceived lack of compassion for the students:

“Professors, especially, some have been very lenient, and kind during this. But I've heard others struggle with their professors who, you know, are acting like oh this is just another online class like when it says if this class was online during a normal time they didn't acknowledge the pandemic, or how students were doing. And this was a challenge for a lot of students, because they're like I can't get my work done and they (professors) answer ‘Okay. Oh well'.” (Female, White, Undergraduate, Wave 2 Interview #5).

#### Suggestions for Improving Mental Health Services Within the University Community

After in depth exploration of their experiences and attitudes, students were asked to give suggestions for ways in which the university could improve its response to mitigate further mental health impacts due to the pandemic. Suggestions to improve students' mental health included one student suggested receiving “more days off” as well as having designated “spaces” to give students a chance to “get out of their houses” (Female, Hispanic/Latinx, Undergraduate, Wave 2 Interview #2). For recommendations regarding how to improve communication regarding mental health one student suggested for the school to “consolidate” the emails so that they are “easier to read.” They also suggested making the emails “pop out” if they contain important information so that students would know to read the message (Female, Asian, Undergraduate, Wave 2 Interview #14).

Other students gave similar recommendations regarding communication and university actions to improve mental health among students.

“Well definitely giving us like more days off like I understand not wanting us to like go out and party and stuff like that but like they're college kids, they're going to do it anyways but just like maybe just like being like hey we see you, we understand, it's hard. We'll try to like make this better or like, give us spaces so like the library wasn't even open until like a few weeks ago and it's like that could have been a great space for people to like be able to get out of their house.” (Female, Hispanic/Latinx, Undergraduate, Wave 2 Interview #2).

## Discussion

The past year has brought on unique changes and challenges to the lives of university students, staff and administrators. In the midst of the pandemic students had to figure out how to adjust to online learning, social isolation and stressors that arise due to the pandemic. This study aimed to develop a deeper understanding of the various factors that impacted student mental health and ways in which university faculty and administrators might mitigate future negative impacts. We found that many students experienced some type of toll on their mental health through loneliness, anxiety, precarity, frustration and prior mental health problems. A previous study conducted by Debowska et al. (7) looked at university students' stress, depression, anxiety and suicidality at the beginning of the pandemic in Poland. The results of their study show that there was a significant increase in depression as the pandemic progressed. It was found that social distancing and isolation which is believed to have led to increased loneliness and contributed to the development of the symptoms of depression. The study also found increased levels of stress and anxiety in the study population. Another study looking into the implication of college campus relocation due to COVID-19 on student mental health found that mandated relocation led to more “COVID-19 related grief, loneliness and generalized anxiety symptoms.” (28). These results were mirrored in the responses that students gave for this study. Relocating from campus increased stress as well as isolation, placing a toll on student mental health. Related studies that have looked at the impact of COVID-19 and changes to college campuses have shown that the pandemic has led to a general decline in mental health among college students (9, 10, 29–31). The decline in mental health among university students coupled with a potential commensurate decline in health-seeking behaviors (32) raises serious concerns about a co-pandemic of poor mental health.

The current study illuminated sources of stress experienced by students at a large U.S. university during the pandemic. Although academic stress was one source, many students had compounding stressors throughout the pandemic such as fear for the safety of their family, big-picture sources of stress, no longer communicating with friends and stress from dealing with life during a pandemic and the societal changes that accompanied COVID-19. Previous studies looking at university students' anxiety after COVID-19 found similar results. Wenjun et al. (6) found that students who had relatives or friends with COVID-19 had increased anxiety. Other aspects that were positively correlated with stress symptoms were economic effects, impacts on daily life and academics (6). Identifying specific stressors can help target interventions to mitigate mental health impacts.

Many students who participated in the study had used tools such as self-care, professional health or university resources to support their mental wellbeing. We also learned how students felt about the university's ability to support student mental health and the various ways in which a university might increase its mental health support and outreach in the future. Students in this study highlighted the importance of support from professors. Previous studies have highlighted the importance of providing professors with the proper training and tools to support online learning (33–35). This study suggested two priorities for university administrators' approach to supporting student health that may warrant further exploration. The first is that students may expect the university to have enough resources so that all students can have access to the resources they need and transparency regarding the limits of what a university can offer may help address unmet expectations. The second is the potential need to further study the most effective communication strategies regarding available mental health resources and outreach that emphasizes the importance of mental health and wellbeing. A viewpoint article by Liu et al. (36) highlighted similar priorities to address college students' mental well-being. They found it was important that students have both access to mental health services and that there is outreach to all students targeting low income, underrepresented minorities, and first-generation students.

This study should be considered in the context of its strengths and limitations. The collection of data over a 6-month time period at the start of the second year of the pandemic meant that students were reporting their experiences at a time when uncertainty regarding the pandemic persisted and the duration of isolation may have had increasing mental health impacts. Peer-led focus groups and interviews may have increased the potential for open discussion of difficult subjects. Demographic data were not reported by five participants, which limited the description of the sample and self-identified gender rather than biological sex was asked of participants because of the increasing evidence of higher risk for poor mental health for people in self-identified gender minority groups. Mental health diagnoses were not verified and limits on the reach of university support were not addressed. Although the number of interviews conducted was supported in the literature and theoretical saturation was reached, different disciplines may have different expectations for sample size for this type of interview study. Qualitative study design is most commonly not intended to generate generalizable data and the heterogeneity of state and university-level responses, may mean conducting this study in different settings could identify different themes.

## Conclusions

This study reinforces much of the current literature on the mental health impacts of the COVID-19 pandemic on university students in the U.S. The narrative approach used in this study offers additional insights that may inform studies aiming to increase understanding of how mental health impacts for university students may vary and the role of universities in mitigating these impacts as the pandemic continues to disrupt university students' lives for a third year. Learning more, both qualitatively and quantitatively, about the various impacts on student mental health and the sources of these stressors may enable universities to develop interventions within their scope of practice that target these potential causes of harm and create effective messaging and targeted resources that will help to support students' mental health. Examples of specific university actions that may warrant further study based on the themes identified in this study include providing students with more breaks during the school year than were allowed pre-pandemic and emphasizing the importance of daily self- care and mental wellbeing. Furthermore, this study suggests that studies that evaluate the utility and effectiveness of structured platforms that enable university faculty and administrators to be attuned to the state of the student body's and the effectiveness of specific designed to support student mental health may be warranted.

## Data Availability Statement

The raw data supporting the conclusions of this article will be made available by the authors, without undue reservation.

## Ethics Statement

The studies involving human participants were reviewed and approved by University of Massachusetts Amherst Institutional Review Board. Written informed consent for participation was not required for this study in accordance with the national legislation and the institutional requirements.

## Author Contributions

SG, AL, SP, TS, and JR: conceived the study questions and design. JR, TS, EC, SG, AL, and SP: collected data. JK, JR, TS, EC, SG, AL, and SP: analyzed the data and reviewed the final version. JK: drafted the manuscript. All authors contributed to the article and approved the submitted version.

## Funding

Internal funding was used for participant incentives and student time.

## Conflict of Interest

The authors declare that the research was conducted in the absence of any commercial or financial relationships that could be construed as a potential conflict of interest.

## Publisher's Note

All claims expressed in this article are solely those of the authors and do not necessarily represent those of their affiliated organizations, or those of the publisher, the editors and the reviewers. Any product that may be evaluated in this article, or claim that may be made by its manufacturer, is not guaranteed or endorsed by the publisher.
