# Supplementary material for: Considerations for Meeting Students' Mental Health Needs at a U.S. University During the COVID-19 Pandemic: A Qualitative Study
Source: Front Public Health. 2022 Feb 2;10:815031. doi: 10.3389/fpubh.2022.815031 (PMC8863128; doi:10.3389/fpubh.2022.815031)
Supplement: Supplementary file 2 [file Table_2.DOCX]

**Wave 2 Interview Guide**

**Perceptions, Responses, and Needs Related to the COVID-19 Pandemic: A Narrative Perspective**

Thank you for agreeing to be interviewed about your experiences with the COVID-19 pandemic and aspects of UMass’s response to the pandemic. I first want to check to be sure you received the consent form and if you have any questions for me about the study.

Before we move on to our questions. I just want to remind you that I would like to record this interview, so I don’t miss any of what you have to say. Any part of our conversation that has identifying information will be deleted from the transcript of the recording. Is it okay if I record?

You can also choose not to answer any of the questions I ask and can stop the interview at any time.

1. The pandemic has affected so many people and every person’s experience has been different. Can you tell me a little bit about your experience with the pandemic over the past year?

Probes:

a. Hardest aspects

b. Silver linings, if any

1. What has been your **overall impression** of UMASS’s handling of the pandemic?

Probes:

- - Positives/Negatives
  - Time periods – early (Spring 2020); middle (Fall 2020); most recent (Spring 2021

1. How do you feel about UMASS’s **communication** with students during the pandemic?

Probes:

- - What worked well/less well
  - UMass’s messaging for staying COVID safe
  - Getting tested twice a week
  - UMass’s messaging when it decided to reverse its decision to re-open in Fall 2020
  - UMass’s messaging when students went into the self-sequestering in spring 2021
  - What if anything do you think UMass could have or should have been done differently in encouraging students to stay COVID safe?
  - How do you see UMass’s response in comparison to other universities’ responses?
  - What do you see as pros and cons of how information and policies have been disseminated to students from the administration?
  - What do you think UMass should do going forward to address challenges presented by the pandemic?
  - Recommendations for improvements in communication if any

1. Now I would like to change gears a little bit and ask a few questions about COVID-19 and vaccines.
   - What sources have you relied on most for information about COVID vaccines?
   - What do you think about UMASS’s communication about vaccines?
   - What do you think UMASS’s policy on vaccination should be for the fall (and why)?

Health Information/Direct Experience with COVID

1. The next set of questions is about the pandemic’s effect on student mental health. First, can you share a little bit about how you feel the pandemic has affected your mental health, if at all?

Probes:

- - Sadness/depression
  - Anxiety/worry
  - Loneliness
  - Hopelessness
  - Frustration
  - Whether had mental health challenges prior to the pandemic (emphasize this is common)

1. What are some of the things that have been the greatest source of stress or anxiety for you?
   - . Not being able to see friends
   - Family members at risk
   - Big picture issues like government handling of pandemic
2. What resources have you used to support your mental health during the pandemic? [If none, asked if wanted to or tried to and what got in the way].
   - . Self – exercise, meditation, music, seeing people on Zoom, seeing people in person
   - Therapist/professional – had before or new
   - . Crisis lines

1. How do you feel UMASS has done supporting students’ mental health?
   - What UMASS resources they are aware of/how they learned about them
   - Whether used/tried to use if not covered in #6
   - Thoughts on ways to mitigate impact of remote learning on mental health
   - . Suggestions for what else UMASS could/should do
2. Are there any other things you would like to share about your experience with the university's response COVID-19 pandemic particularly in relation to your mental health and UMass’s COVID-19 messaging?
3. Do you have any questions for me?

Thank you very much. I learned so much from this interview and appreciate you sharing your experiences and thoughts with me.
